# Neighborhood Diversity Is Good for Your Health: An Example of Racial/Ethnic Integration and Preterm Birth in Texas

**DOI:** 10.1007/s40615-024-02117-7

**Published:** 2024-08-13

**Authors:** Shetal Vohra-Gupta, Bethany M. Wood, Yeonwoo Kim, Quynh Nhu La Frinere-Sandoval, Elizabeth M. Widen, Catherine Cubbin

**Affiliations:** 1 Steve Hicks School of Social Work, The University of Texas at Austin, 1925 San Jacinto Blvd, Austin, TX 78712 USA; 2 School of Social Work, The University of Texas at Arlington, 501 W Mitchell St, Arlington, TX 76010 USA; 3 Department of Kinesiology, University of Texas at Arlington, 411 S. Nedderman Drive, Arlington, TX 76019 USA; 4 Department of Nutritional Sciences, The University of Texas at Austin, 1400 Barbara Jordan Blvd, Austin, TX 78723 USA

**Keywords:** Neighborhood diversity, Segregated neighborhoods, Preterm birth, Adverse birth outcomes, Racial disparities

## Abstract

Racial concentration of neighborhoods is often associated with the risk of preterm birth (PTB) for women. This study examined differences between racially diverse and racially concentrated neighborhoods when examining preterm birth. Individual-level data were obtained from Texas natality files for 2009–2011, and neighborhood-level (i.e., census tract) data were obtained from the decennial census in 2010 and the American Community Survey 2005–2009. We used multilevel modeling to assess the association between neighborhood racial diversity and odds of PTB, after controlling for individual characteristics, neighborhood poverty, and population density. We found that neighborhood racial diversity and concentration matter for PTB. Results suggest that systemic racism is still key to understanding PTB. Furthermore, findings support policies that prevent displacement from gentrification of diverse neighborhoods and promote equal access to health-related resources for women in predominantly Black, Hispanic, and/or immigrant neighborhoods.

## Introduction

Preterm birth (PTB), birth before 37 weeks of gestation, is a common adverse birth outcome that can have lasting physical and mental health consequences [1–4]. Specifically, PTB increases the risk of infant mortality [5] and low birth weight which can be harmful to a child’s life expectancy [6]. Nationally, PTB is responsible for two-thirds of infant deaths and half of subsequent childhood neurological problems [7]. It is also associated with high levels of asthma; and this finding has been consistent with the past decade of literature (see, for example, Goyal et al. [8]; Sonnenschein-van Der Voort et al. [9]; Steffensen et al. [10]). In addition, PTB increases the risk of neurocognitive diagnoses, like being diagnosed with autism [11, 12], ADHD [13], and lower IQ levels [14] as well as chronic disease, specifically cardiovascular disease [1, 15]. Over the past decades in the USA, the overall prevalence of adverse birth outcomes has been declining; however, substantial and persistent racial and ethnic disparities in adverse birth outcomes remain [16].

The risk of PTB for most people of color (POC) is disproportionality high in the USA where the percentage of PTB to non-Hispanic Black women is about 50% higher, non-Hispanic American Indian/Alaska Native or Native Hawaiian/Other Pacific Islander women is about 30% higher, and to Hispanic women is 7% higher, compared with non-Hispanic White women; non-Hispanic Asian women have about 3% lower rates, however, than non-Hispanic White women [17]. A key structural determinant of PTB is racial and ethnic residential segregation and the adverse neighborhood conditions that accompany these neighborhoods [18–21]. Racial/ethnic segregation increases racial inequalities in social and economic factors and thus has consequences for the health and well-being of individuals [22, 23], including PTB.

### Theoretical Frameworks

Ecosocial theory [24], along with social determinants of health and neighborhood effects frameworks, provide conceptual models for this study. Key to ecosocial theory is the embodiment, or the ways in which social context influences physical health. Neighborhood environment, as one of these social contexts—encompassing social, service, and physical environments—has long been recognized as a social determinant of health [25–27]. Many studies have found that neighborhood context, inclusive of factors like greenspace and poverty, can adversely impact mental and physical health across the lifespan [28–30]. Neighborhood characteristics, such as a lack of resources or assets, are embodied through increased stress that can be associated with many adverse birth consequences, like PTB [21, 31–36]. Living in a low-income neighborhood is also associated with a high risk of stress [37] and with a higher risk of adverse birth outcomes [38, 39]. The relationships between neighborhood characteristics and PTB also have intersectional variations; for example, one study found that for Black women, living in gentrified neighborhoods was associated with higher PTB, whereas White women living in the same type of neighborhoods had a lower risk of PTB [40].

The connecting piece between neighborhood formation and health disparities lies within the history of US policy. Legislation, like the Housing Act of 1949, allowed politicians to target communities of color through forced eviction to remove families from their neighborhoods [41]. Spatial discrimination practices, like redlining, are discriminatory actions that separate areas by racial lines; these practices are associated with high unemployment and other disparities in communities of color [42]. Although the 1940s practice of redlining is an injustice of the past, the remnants of physical barriers, like highways, continue to separate neighborhoods by race [43]. Additionally, current gentrification disproportionately displaces POC [44], who are far more likely to live in low-income neighborhoods than their White counterparts [45–47]. Furthermore, racially segregated neighborhoods are associated with lower-quality public services, lower educational attainment, higher rates of police misconduct, and higher mortality [48–52]. Massey [53] argues that racism and residential segregation are, in fact, the key predictors that explain the impacts of disparities among the Black community. Therefore, neighborhood racial environments, as a determinant of health, along with education, career, family, and social outcomes, have been studied at length; however, these relationships are often not studied with a racial composition lens that includes racially diverse neighborhoods, which is important given the increasing current and projected demographic diversity of the USA.

Studies have highlighted the influence of ethnic density, racial discrimination, and perceptions of neighborhood quality on PTB risk [54–56]. Additionally, racial composition and residential duration, or how long one lives in the neighborhood, have been found to modify the risk of PTB. These findings emphasize the importance of considering the racial, ethnic, and diverse makeup of neighborhoods in which pregnant individuals reside, as these factors can impact birth outcomes. Theoretically, ecosocial theory and neighborhood effects frameworks contribute to the importance of why diverse neighborhoods, compared with racially and ethnically concentrated neighborhoods, need to be studied when investigating PTB. Ecosocial theory emphasizes interconnections between social, economic, and environmental factors and their influence on health outcomes. Neighborhood effects theory states characteristics and resources of a neighborhood influence the health and well-being of its residents. Both theories highlight the potential significance of studying diverse neighborhoods, specifically how factors such as cultural amenities, economic opportunities, and social networks and their collective interactions with race and ethnicity contribute to nuanced health disparities. Health interventions including policy and practice must be tailored to the unique needs and characteristics of different communities. A study that examines both diverse and racially and ethnically concentrated neighborhoods fills a gap by working to identify commonalities and differences, enabling the development of targeted interventions and offering a deeper understanding of the root causes of disparities.

The present study addresses the previous gaps in the literature by examining the relationship between multiple categories of neighborhood racial/ethnic diversity on PTB and whether racially/ethnically diverse neighborhoods help “explain” racial inequities in PTB, in Texas, a large and diverse state. This study purports the following hypotheses: in neighborhoods characterized by racial and ethnic diversity, PTB outcomes are expected to be lower compared to more racially/ethnically concentrated neighborhoods, yet higher compared to predominantly White neighborhoods. Conversely, in predominately White neighborhoods, the study anticipates the lowest PTB outcomes among all other neighborhood types.

## Materials and Methods

### Data

Individual-level data were obtained from natality files for all live, singleton births for 2009–2011 in Texas, which includes information on birth outcomes, maternal sociodemographic characteristics, and paternal educational level. Neighborhood-level (i.e., census tract) data were obtained from the decennial census in 2010 and the American Community Survey 2005–2009. We linked the individual-level data to the neighborhood-level data based on geocodes for women’s residential addresses on birth certificates.

Our analytic sample included all singleton births to Black (non-Hispanic), Hispanic, White (non-Hispanic), and “Other” women (a category provided by Texas vital records to include a combination of all other groups such as American Indians/Alaskan Natives, Asian and Pacific Islanders, and multiple race groups) after first excluding records missing birth weight, those with gestational age < 22 or > 44 weeks, and those with biologically implausible combinations of birth weight and gestational age, *N* = 1,040,642 births in 5196 tracts [57]. We then excluded those with missing data on covariates, except for missing paternal education and prenatal care initiation, where we included a separate category because of the relatively high amount of missing data (15% and 6%, respectively), resulting in a final sample of 1,037,341.

### Individual Measures

Our outcome variable is PTB, defined as fewer than 37 weeks of completed gestation based on the birth certificate estimate of gestational age (otherwise full-term birth). Individual-level covariates included maternal age, race/ethnicity, parity, marital status, educational level, prenatal care initiation, and paternal educational level.

### Neighborhood Measures

**Fig. 1:**
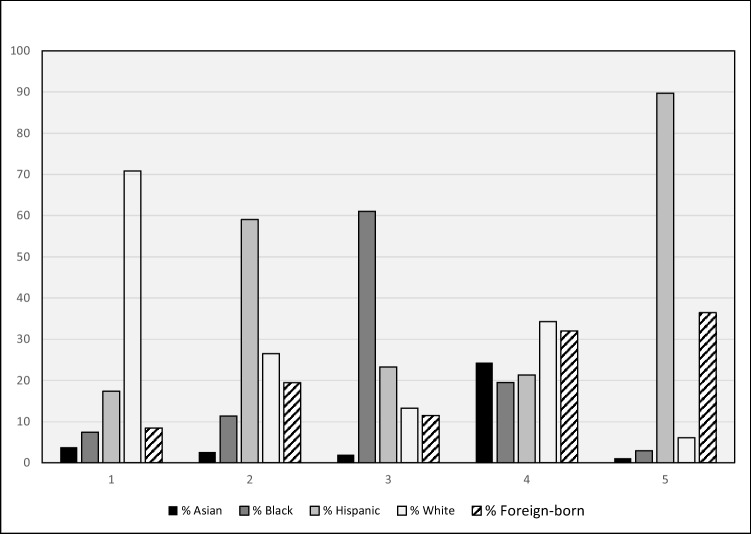
Neighborhood cluster characteristics, *N* = 5196 census tracts, Texas

Our main exposure variable is neighborhood racial/ethnic diversity. We conducted a cluster analysis with five variables (% Asian, Black, Hispanic, White, and foreign-born) for all census tracts in Texas. The scree plot suggested two to six expected clusters, and we examined the five and six cluster results (means and sample sizes) so that we could distinguish between more than only a few neighborhood diversity types that would be available with two to four clusters. One of the clusters for the six cluster results had very few tracts (53, or 1%) so we decided to use the five cluster results in our analyses. Based on the variable means in the cluster analysis, we categorized census tracts into five types of neighborhood racial/ethnic diversity: (1) *White*, (2) *Hispanic*, *White & Immigrant*, (3) *Black & Hispanic*, (4) *Diverse*, and (5) *Hispanic & Immigrant*. Using the variable means to illustrate, *White* tracts had 71% White persons on average and 4–18% of other groups, while the *Diverse* tracts had 24% Asian, 20% Black, 21% Hispanic, 34% White, and 32% foreign-born persons on average (Fig. 1).

Neighborhood-level covariates were neighborhood poverty and population density. The latter was operationalized as population per square mile as a proxy of urbanization and was log-transformed because of non-normality.

### Statistical Analysis

We first examined the prevalence of preterm birth by maternal race/ethnicity and neighborhood diversity cluster (Table 1) as well as the distribution of the variables (Table 2). We then estimated hierarchical generalized linear models (HGLM) to examine the association between neighborhood racial/ethnic diversity and PTB (Table 3). Hierarchical modeling was conducted because the data were clustered by census tract, even though the intra-class correlation coefficient (ICC) was low (0.02). We first estimated bivariate unadjusted associations between each variable and PTB. The second model included individual characteristics (age, race/ethnicity, marital status, parity, education levels, prenatal care initiation), testing for their associations with the risk of PTB. The third model added population density and neighborhood poverty to the second model. The fourth model added neighborhood racial/ethnic diversity (the reference group was *White* neighborhoods) to the third model. We also estimated the fourth model by using other types of neighborhood racial/ethnic diversity as the reference group (Table 4). Throughout the modeling process, − 2 log-likelihood, the Akaike information criterion, and the Bayesian information criterion were assessed for model fit. We used SAS software version 9.4 for all analyses.

**Table 1:** Sample sizes and prevalence of preterm birth, natality files from Texas, Texas, USA, 2009–2011, *N* = 1,037,341

	Total	Neighborhood cluster				
		*White*	*Hispanic, White, & Immigrant*	*Black & Hispanic*	*Diverse*	*Hispanic & Immigrant*
# mothers	1,037,341	433,313 42%	337,439 33%	73,567 7%	40,664 4%	152,358 15%
# census tracts	5196	2665 51%	1458 28%	359 7%	167 3%	547 11%
Preterm birth rate (%)	10.9	9.5	11.7	12.9	9.9	12.6
Black women	14.6	13.9	14.9	15.3	13.2	15.5
US-born Hispanic women	12.3	10.6	12.7	11.3	9.8	13.4
Immigrant Hispanic women	10.7	9.9	10.4	10.5	9.5	11.8
White women	9.0	8.6	10.2	9.5	8.7	11.6
Other women	9.4	8.9	10.7	10.4	8.9	12.3

**Table 2:** Sample characteristics, natality files from Texas, Texas, USA, 2009–2011, *N* = 1,037,341

Characteristics	Number	% or mean (range)
Mother’s age		
11–19 years of age	129,861	12.5
20–34 years of age	783,652	75.3
35 or above	127,124	12.2
Mother’s race/ethnicity		
Black, non-Hispanic	119,461	11.5
Hispanic, US-born	276,913	26.6
Hispanic, immigrant	236,029	22.7
White, non-Hispanic	355,809	34.2
Other	52,380	5.0
Parity		
First child	412,371	39.6
Second-fourth child	582,672	56.0
Fifth child or more	45,489	4.4
Mother’s marital status		
Married	598,065	57.5
Not married	442,557	42.5
Mother’s education level		
Less than high school	263,674	25.4
High school/GED	280,290	27.0
Some college	272,538	26.2
College graduate or more	223,203	21.5
Father’s education level		
Less than high school	217,193	20.9
High school/GED	257,074	24.7
Some college	218,556	21.0
College graduate or more	196,171	18.9
Missing	151,648	14.6
Mother’s prenatal care initiation		
First trimester care	604,385	58.1
Delayed/no prenatal care	372,824	35.8
Missing	63,433	6.1
Neighborhood population density (persons per square mile)	5196 tracts	3390.8 (0.3–55,254.8)
Neighborhood poverty (% below poverty level)	5196 tracts	13.2 (0.0–95.0)

**Table 3:** The association between neighborhood racial/ethnic diversity and preterm birth, natality files from Texas, Texas, USA, 2009–2011, *N* = 1,037,341

	Model 1		Model 2		Model 3		Model 4	
	OR	95% CI	OR	95% CI	OR	95% CI	OR	95% CI
*Fixed effects*								
Mother’s age								
12–19 years of age	1.22^***^	1.20–1.24	1.04^***^	1.02–1.06	1.04^***^	1.02–1.06	1.04^**^	1.02–1.06
20–34 years of age	1.00		1.00		1.00		1.00	
35 or above	1.30^***^	1.27–1.32	1.37^***^	1.35–1.40	1.38^***^	1.35–1.41	1.38^***^	1.35–1.41
Mother’s race/ethnicity								
Black, non-Hispanic	1.70^***^	1.67–1.74	1.50^***^	1.47–1.53	1.48^***^	1.45–1.52	1.48^***^	1.44–1.51
Hispanic, US-born	1.34^***^	1.32–1.36	1.18^***^	1.16–1.20	1.16^***^	1.14–1.18	1.13^***^	1.11–1.15
Hispanic, immigrant	1.16^***^	1.14–1.18	0.96^***^	0.94–0.98	0.95^***^	0.93–0.97	0.92^***^	0.90–0.95
White, non-Hispanic	1.00		1.00		1.00		1.00	
Other	1.07^***^	1.04–1.11	1.13^***^	1.10–1.17	1.14^***^	1.10–1.17	1.13^***^	1.09–1.17
Mother’s marital status								
Married	1.00		1.00		1.00		1.00	
Not married	1.28^***^	1.27–1.30	1.09^***^	1.08–1.11	1.09^***^	1.07–1.11	1.09^***^	1.07–1.11
Parity								
First child	1.00		1.00		1.00		1.00	
Second-fourth child	1.04^***^	1.03–1.06	1.05^***^	1.03–1.06	1.04^***^	1.03–1.06	1.04^***^	1.03–1.06
Fifth child or more	1.61^***^	1.55–1.68	1.40^***^	1.35–1.47	1.39^***^	1.33–1.45	1.39^***^	1.33–1.45
Mother’s education level								
Less than high school	1.58^***^	1.55–1.62	1.36^***^	1.32–1.40	1.34^***^	1.30–1.38	1.34^***^	1.30–1.38
High school/GED	1.42^***^	1.39–1.45	1.22^***^	1.19–1.25	1.20^***^	1.17–1.23	1.20^***^	1.17–1.23
Some college	1.31^***^	1.28–1.34	1.15^***^	1.12–1.18	1.14^***^	1.11–1.17	1.14^***^	1.11–1.17
College graduate or more	1.00		1.00		1.00		1.00	
Father’s education level								
Less than high school	1.51^***^	1.48–1.55	1.28^***^	1.25–1.32	1.26^***^	1.23–1.30	1.26^***^	1.22–1.29
High school/GED	1.45^***^	1.41–1.48	1.26^***^	1.23–1.30	1.25^***^	1.21–1.28	1.24^***^	1.21–1.27
Some college	1.30^***^	1.27–1.33	1.19^***^	1.16–1.22	1.18^***^	1.15–1.21	1.17^***^	1.14–1.20
College graduate or more	1.00		1.00		1.00		1.00	
Missing	1.85^***^	1.81–1.89	1.46^***^	1.42–1.51	1.44^***^	1.40–1.48	1.43^***^	1.40–1.48
Mother’s prenatal care initiation								
First trimester care	1.00		1.00		1.00		1.00	
No first trimester care	0.94^***^	0.93–0.95	0.84^***^	0.83–0.85	0.84^***^	0.83–0.85	0.84^***^	0.83–0.85
Missing	1.50^***^	1.46–1.53	1.43^***^	1.39–1.46	1.42^***^	1.39–1.45	1.41^***^	1.38–1.45
Neighborhood population density (logged)	1.01	1.00–1.01			0.99^***^	0.99–1.00	0.98^***^	0.98–0.99
Neighborhood poverty (per 10% increase)	1.10^***^	1.10–1.10			1.04^***^	1.03–1.04	1.02^***^	1.01–1.03
Neighborhood diversity								
*Hispanic, White, & Immigrant*	1.27^***^	1.25–1.29					1.10^***^	1.08–1.13
*Black & Hispanic*	1.44^***^	1.40–1.49					1.05^**^	1.01–1.08
*Diverse*	1.04	0.99–1.09					1.03	0.99–1.08
*Hispanic & Immigrant*	1.38^***^	1.35–1.42					1.16^***^	1.12–1.19
*White*	1.00						1.00	
*Random effects*								
Level-2 intercept	–		0.0182		0.0169		0.0157	
Model fit								
− 2 log-likelihood	–		707,743		705,579		705,463	
Akaike information criterion	–		707,783		705,623		705,512	
Bayesian information criterion	–		707,914		705,767		705,686	

Note. Model 1 indicates a bivariate unadjusted model. Model 2 includes individual-level variables. Model 3 includes population density and neighborhood poverty trajectories in addition to model 2. Model 4 includes neighborhood diversity in addition to model 3

*OR*, odds ratio; *CI*, confidence interval

^*^p < .05, ^**^p < .01, ^***^p < .001

**Table 4:** The association between neighborhood racial/ethnic diversity and preterm birth with different reference groups, natality files from Texas, Texas, USA, 2009–2011, *N* = 1,037,341

	OR	95% CI
**[Reference = *Hispanic, White, & Immigrant* neighborhoods]**		
*Black & Hispanic*	0.95^**^	0.92–0.98
*Diverse*	0.94^**^	0.90–0.98
*Hispanic & Immigrant*	1.05^*^	1.03–1.08
*White*	0.91^***^	0.89–0.93
**[Reference = *Black & Hispanic* neighborhoods]**		
*Hispanic, White, & Immigrant*	1.05^**^	1.02–1.08
*Diverse*	0.99	0.94–1.04
*Hispanic & Immigrant*	1.10^***^	1.06–1.14
*White*	0.95^**^	0.92–0.99
**[Reference = *Diverse* neighborhoods]**		
*Hispanic, White, & Immigrant*	1.06^*^	1.01–1.10
*Black & Hispanic*	1.01	0.96–1.06
*Hispanic & Immigrant*	1.11^***^	1.06–1.16
*White*	0.96	0.92–1.00
**[Reference = *Hispanic & Immigrant* neighborhoods]**		
*Hispanic, White, & Immigrant*	0.95^***^	0.93–0.98
*Black & Hispanic*	0.91^***^	0.88–0.94
*Diverse*	0.90^***^	0.86–0.94
*White*	0.87^***^	0.84–0.89

Note. All covariates included in Model 4, Table 3 were adjusted

*OR*, odds ratio; *CI*, confidence interval

^*^p < .05, **p < .01, ***p < .00

## Results

Table 1 presents sample sizes and prevalence of PTB overall and for each neighborhood cluster. The prevalence of PTB was 15% among Black women, 12% among US-born Hispanic women, 11% among foreign-born Hispanic women, and 9% among White and Other women. Overall, PTB rates were lowest in *White* and *Diverse* neighborhoods; PTB rates for Black and Hispanic women were lowest in *Diverse* neighborhoods, whereas PTB rates for White and Other women were lowest in *Diverse* and *White* neighborhoods. Slightly over half the neighborhoods were classified into the *White* cluster, only 3% were classified into the *Diverse* cluster, and 7–28% were classified into the other three clusters.

**Fig. 2:**
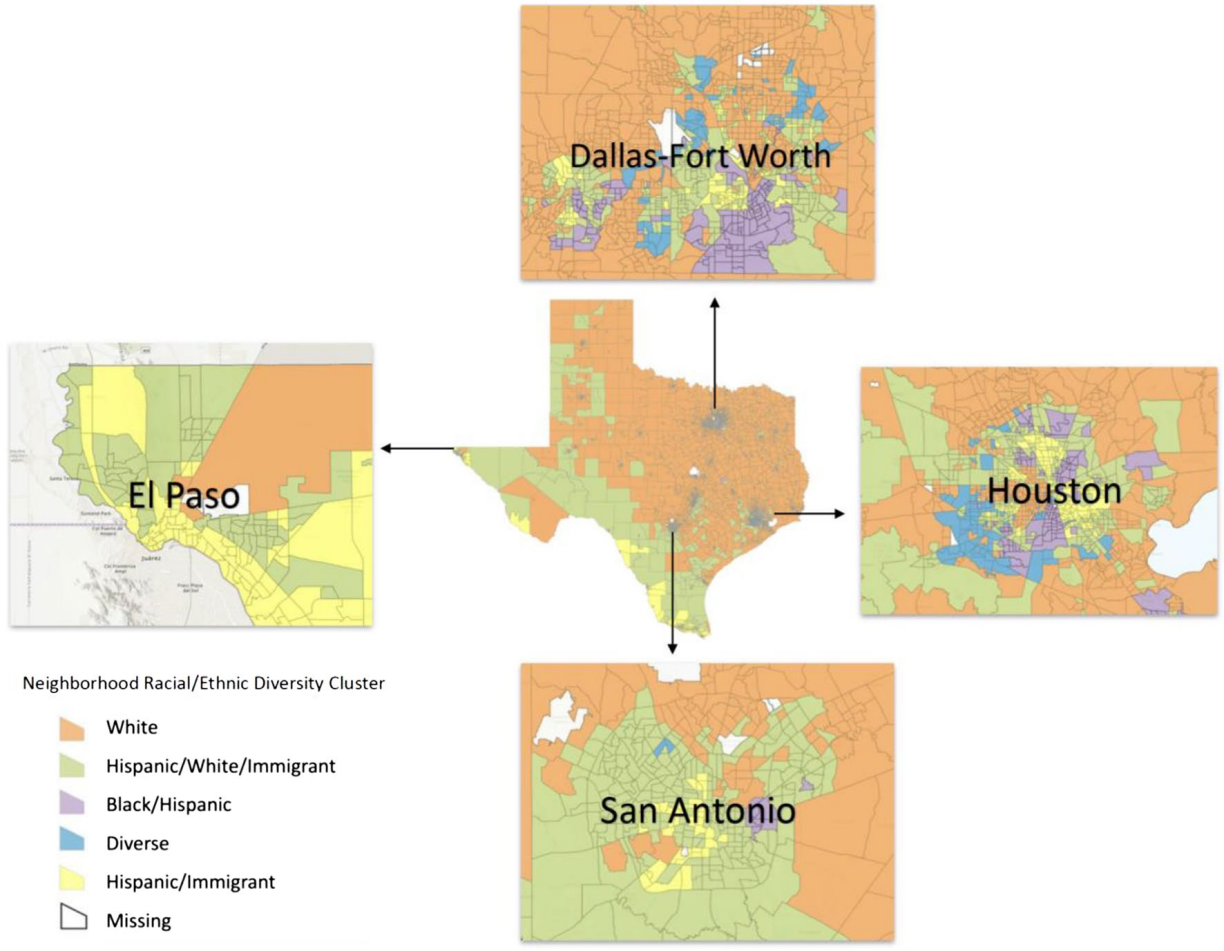
Neighborhood racial/ethnic diversity clusters by census tract, Texas

Figure 2 shows the categorized census tracts throughout Texas with highlights of Dallas-Fort Worth, Houston, San Antonio, and El Paso. When looking at the entire state, it appears that census tracts along the Texas-Mexico border tended to have more of the *Hispanic, White & Immigrant* cluster neighborhoods whereas central and northeast Texas had more *White* cluster neighborhoods. More patterns emerge when examining the highlighted cities. The clusters of *Diverse* neighborhoods were most prevalent in the urban metropolitan areas of Dallas-Fort Worth and Houston, whereas rural areas tended to be comprised of the *White* and *Hispanic, White & Immigrant* clusters. Dallas-Fort Worth and Houston also had the most tracts comprised of the *Black & Hispanic* cluster. Finally, El Paso had more of the *Hispanic & Immigrant* cluster than the other highlighted cities (see Fig. 2).

Covariate characteristics are presented in Table 2. Three-quarters of women were 20–34 years old, nearly half were Hispanic, two-fifths were primiparous, and 58% were married. There was a fairly even distribution of births by maternal and paternal education but with 15% reporting missing information on paternal education, and over one-third were to mothers without first-trimester prenatal care. Average population density was about 3400 people per square mile, and on average, 13% of people in their neighborhoods had incomes below the poverty level.

Between-neighborhood variance in PTB decreased from 0.0505 in an intercept-only model (or “null” model) to 0.0157 after considering individual characteristics, population density, neighborhood poverty, and neighborhood racial/ethnic diversity, and model 4 had the best model fit based on − 2 log-likelihood, AIC, and BIC values (Table 3). The unadjusted association between neighborhood diversity and PTB (Model 1) demonstrates that living in neighborhoods characterized as (1) *Hispanic, White, & Immigrant*, (2) *Black & Hispanic*, or (3) *Hispanic & Immigrant* was associated with 27 to 44% higher odds of PTB compared with living in *White* neighborhoods while living in a *Diverse* neighborhood was associated with similar odds of PTB compared with living in *White* neighborhoods. These increased odds remained significant but attenuated (5–16% higher odds) in the model adjusted for individual- and neighborhood-level covariates (Model 4). All other covariates remained significant in the fully adjusted model in expected directions, except for prenatal care initiation, where women who did not initiate care in the first trimester had lower odds compared with those who initiated care in the first trimester.

Table 4 presents neighborhood diversity results from the fully adjusted model in Table 3, Model 4, except substituting in different reference groups. The results demonstrate that *Hispanic & Immigrant* neighborhoods confer the most disadvantage in that living in any other neighborhood diversity category has 5–13% lower odds of PTB compared with living in *Hispanic & Immigrant* neighborhoods (panel D) and, conversely, living in *Hispanic and Immigrant* neighborhoods has 5–11% higher odds of PTB compared to other neighborhood diversity categories.

## Discussion

The goal of this study was to explore the relationships between racially/ethnically diverse neighborhoods and PTB. We advance previous work by investigating whether residing in a particular type of neighborhood impacted the odds of experiencing PTB after extensive controls for individual- and neighborhood-level confounding factors. We found that over and above neighborhood poverty and population density and individual maternal race/ethnicity and education, neighborhood diversity mattered for PTB in both positive and negative ways depending upon the racial/ethnic/nativity makeup of the neighborhood. Living in *Diverse* neighborhoods offered potential advantages that were similar to living in *White* neighborhoods, and living in *Hispanic & Immigrant* neighborhoods experienced greater potential disadvantages over each of the other neighborhood clusters.

A closer examination of racially/ethnically concentrated neighborhoods as well as racially/ethnically diverse neighborhoods is warranted as a strategy to decrease the prevalence of PTB, as our findings indicate. The physical separation of race/ethnicity in certain neighborhoods is an institutional mechanism enforced by policy and systems of overt and covert discrimination [23]. In fact, more racial/ethnic segregation over time builds the concentration of disadvantage within these neighborhoods [58], such as having less access to employment opportunities, high-quality public education, nutritious food, mental health care, and medical care [59–64]. Additionally, White flight (movement of White residents from neighborhoods when minority populations move into that neighborhood) and housing discrimination mean that disadvantages for Hispanic and Black neighborhoods continue to exist [65, 66]. These conditions differ when compared to predominantly White and diverse neighborhoods [67], although the latter can be impacted by gentrification-induced displacement. Our descriptive findings imply that for people of color, individual race/ethnicity has less impact on the prevalence of PTB when residing in racially/ethnically diverse neighborhoods. Further research should investigate the factors in *Diverse* neighborhoods that promote a lower risk of PTB. And interventions and policies should prioritize the needs of those living in racially/ethnically segregated neighborhoods (especially those characterized as *Hispanic & Immigrant*) by linking them to adequate resources to reduce stress and improve health outcomes.

### Potential Advantages of Living in Diverse Neighborhoods

In this study, residing in *Diverse* neighborhoods, made up of 24% Asian, 20% Black, 21% Hispanic, 34% White, and 32% foreign-born persons on average, provided similar advantages to living in predominately *White* neighborhoods when it came to reducing the likelihood of experiencing PTB. However, only 3% of all Texas census tracts were *Diverse* (and 4% of births happened within them), compared with 51% of all Texas census tracts that were *White* (and 42% of births happened within them). A large body of literature has pointed to the health inequities that exist in the USA for Hispanic and Black communities due to social determinants of health [68–72]. However, research on racially/ethnically diverse neighborhoods has yielded mixed findings. Some scholars, observing patterns of White flight and gentrification, argue that such neighborhoods are often transitional, shifting dominance from one racial or ethnic group to another [73]. However, other research has shown racially/ethnically diverse neighborhoods represent a long-term social pattern and these neighborhoods exist outside of transitioning gentrifying neighborhoods [73–75]. These communities present substantial benefits and generally afford greater access to resources. For example, a study conducted on racially diverse suburbs in the 50 largest metropolitan areas concluded that populations who move to racially/ethnically diverse suburbs from central city neighborhoods are afforded potential benefits such as better educational opportunities, better access to regional job markets, and a safer living environment [76]. An increase in the immigrant population to a neighborhood contributes to the greater economic development of that area due to a cheaper labor supply, establishing small businesses and contributing to the tax base [77]. Beyond resource availability and economic development, having an area of concentrated advantages tends to exhibit lower allostatic load or chronic stress levels for those living in these communities [76]. Moreover, there is also an overall appreciation for diversity. Greater access to healthcare services, better schools, healthier food options, and access to job opportunities, along with less exposure to air pollution and crime, may help in lowering stress and PTB outcomes. This further strengthens the notion of how social determinants, or the non-medical factors of where one lives, works, plays, and ages, are critical to health and well-being.

### Potential Disadvantages of Living in Hispanic & Immigrant Neighborhoods

When compared with all other clustered neighborhoods, people living in *Hispanic & Immigrant* neighborhoods experienced the highest odds of PTB even when poverty levels were held constant, indicating potential birth outcome disadvantages for residents. Therefore, neighborhood effects may explain the disadvantages identified in this study.

The prevalence of adverse birth outcomes among Hispanic and Black communities in the USA is largely attributed to the history of racial discrimination and social, economic, and political injustice. A previous study showed that Mexican immigrants living in Hispanic-concentrated neighborhoods were more likely to have negative birth outcomes compared to those living in less Hispanic-concentrated neighborhoods [78]. Our findings indicate the impact of both structural and individual racism that *Hispanic & Immigrant* communities may face in the USA. While interaction effects were beyond the scope of this study, future research should examine neighborhood racial/ethnic diversity effects on specific racial/ethnic groups, including Hispanic immigrants and non-immigrants.

Specific to this study, Hispanic neighborhoods in Texas have been shaped by both historical and contemporary discriminatory housing practices, including redlining and gentrification [79]. Literature on Texas indicates that residents who perceive their neighborhood as gentrifying are more likely to report chronic health conditions [80]. Predominantly, Hispanic neighborhoods in Texas have continued to gentrify over the past decades, leading to the displacement and dissolution of many Hispanic Texas neighborhoods [81]. The potential loss of one’s community is associated with increased psychological distress [82–84]. Additionally, historically redlined neighborhoods may present built environmental risks that non-redlined neighborhoods do not face [85–87].

The built environment, inclusive of old housing stock, limited green spaces, pollutant exposure, and proximity to environmental hazards, may pose a risk of PTB for *Hispanic & Immigrant* neighborhoods. Communities that are historically redlined are often adjacent to overpasses and freeways, which exposes residents to hazardous levels of pollution [88]. Further, these neighborhoods often lack tree coverage and green spaces and instead are concrete-dense—combined with climate change, this creates dangerous urban heat islands [85, 89]. A preliminary study demonstrates this is true in Texas Hispanic communities as well [90]. A systematic review of over 32 million US births reported that in over 80% of the studies, heat and air pollution were associated with adverse birth outcomes [88]. Furthermore, predominantly Hispanic neighborhoods frequently face high rates of poverty, and Hispanic households tend to reside in older structures compared to their white counterparts [91, 92]. Neighborhoods with older housing stock often contain environmental pollutants, including lead contamination in water and lead-based paint in homes and buildings. Lead exposure, specifically, is strongly linked with an increased risk of PTB [93–95]. A population-level study of Texas births demonstrated that mothers who lived in high-poverty neighborhoods with older housing built before the 1970’s ban on lead-based paint had a higher incidence of PTB compared to mothers in other areas [96].

Other neighborhood-level effects include limited access to healthcare services, which may be compounded by language barriers, immigration status, and a lack of culturally competent care, which may hinder timely and adequate prenatal interventions [79, 97]. Future studies should assess the impact of documentation status and nativity on healthcare utilization in Texas on adverse birth outcomes, especially given that a recent study reported that a lack of health insurance was the strongest predictor of healthcare non-utilization among Hispanic workers in southwest Texas [98]. Moreover, fear and distress about immigration policies are particularly salient for *Hispanic & Immigrant* neighborhoods. In recent decades, Texas Immigration and Customs Enforcement (ICE) has detained migrants at one of the highest rates in the country [99]. Policies that criminalize immigration are linked to an amplified risk of PTB for women of color [100], and sociopolitical stressors, inclusive of antagonistic political rhetoric and hate crimes, are associated with increased risk of PTB among Latina mothers [101, 102]. Additionally, *Hispanic & Immigrant* communities in Texas may face numerous psychological stressors, including discrimination and acculturation stress, which pose a risk of chronic stress during pregnancy and are associated with higher PTB rates [103].

### Ecosocial theory and additional implications

The findings of this study are consistent with ecosocial theory, specifically in that a neighborhood’s racial/ethnic composition is significantly associated with PTB. Our findings suggest that racial/ethnic composition, accounting for neighborhood poverty, might represent different routes of how stress becomes physically embodied, leading to PTB outcomes (Krieger, 1994). One study that examined highly segregated neighborhoods compared to low segregated neighborhoods found that women living in highly segregated neighborhoods had a significantly higher rate of PTB. This pattern persisted even after accounting for medical and family history [104]. Although POC generally have a higher risk of PTB, our study aligns with ecosocial theory in that the risk may be further exacerbated for Texas residents of predominantly *Hispanic & Immigrant* neighborhoods. These neighborhoods’ disadvantages—inclusive of structural racism in housing policies (e.g., redlining), environmental hazards (e.g., lead-based paint), sociopolitical rhetoric (e.g., criminalizing immigration policies), and limited healthcare access—are stressors that become physically embodied onto individual residents. Based on this finding, there first is a need for research that identifies the highest-risk factors to PTB in these neighborhoods to mitigate the potential birth risks that mothers residing in predominantly *Hispanic & Immigrant* neighborhoods face. Secondly, place-based interventions should target the highest-risk factors of PTB, similar to interventions that have reduced lead exposure (e.g., stripping lead-based paint in older homes) in neighborhoods [105]. Furthermore, implementation of policies that promote racial/ethnic integration in neighborhoods and equitable access to resources through strengthening and enforcing fair housing laws, incentivizing mixed-income housing developments (with enforcement of penalties for violations), and investment in affordable housing may mitigate the PTB impact of neighborhood racial/ethnic stratification. Additionally, policy initiatives that improve neighborhood infrastructure and environmental regulations (e.g., pollution control policies, increasing green spaces, conducting regular health assessments of the built environment) are necessary. Finally, expanding access to quality education and job training programs, providing childcare subsidies and transportation subsidies, and increasing access to affordable healthcare are interventions and policies that may mitigate the impact of neighborhood poverty on PTB.

In our models, most covariates (mother’s age, race/ethnicity, marital status, parity, education, and father’s education) were significant in the directions expected. However, our findings around prenatal care initiation were puzzling. Despite the established prevalence of PTB being lower among women who initiate prenatal care in their first trimester, our models found that the odds of PTB were lower for those who initiated care after the first trimester compared with those who initiated care in the first trimester. While beyond the scope of this paper, future research needs to examine whether this unexpected finding is specific to Texas.

While we conducted correlational analysis, we suggest potential causal relationships between racial/ethnic segregation and PTB: lack of educational and economic opportunities which can increase chronic stress and limit access to health care; socioeconomic conditions which can increase chronic stress and impact health status; environmental stressors such as poor housing conditions or pollution leading to physical and mental health issues that can impact pregnancy; limited access to prenatal and postnatal care; and community support which can provide emotional and psychological support. These should be examined as causal mechanisms to truly impact PTB.

Despite the strengths of a large, diverse population and generalized hierarchical linear modeling, the study has a number of limitations that deserve mention. We could not capture women’s residential mobility before or during the pregnancy as geocoded data were based on residential address at the time of delivery. Additionally, we were also limited by a lack of information on individual-level income or experiences of discrimination which may be important for PTB but are not available on birth records. We examined racial/ethnic/nativity concentrations based on cluster analysis. Future research should consider other ways of measuring the racial/ethnic/nativity makeup of neighborhoods. More research is also needed for additional racial and ethnic groups, for example, Indigenous people living in tribal communities (e.g., Alabama-Coushatta tribe), Asian and Pacific Islanders, and multiracial populations. This study featured a cross-sectional design, and therefore, future research should examine these patterns across time, including how neighborhood racial/ethnic diversity characteristics themselves have changed over time.

## Conclusions

Our work highlights how the racial/ethnic composition of neighborhoods matters in that those who live in predominately *Hispanic & Immigrant* experience higher chances of PTB compared to all other neighborhood types, even after controlling for neighborhood poverty and urbanization. Women giving birth in *Diverse* neighborhoods, in contrast, have similar, low chances of PTB compared with women giving birth in predominantly *White* neighborhoods. This research provides further evidence that systemic racism plays out at the neighborhood level. Embodiment or the absorption of one’s social and environmental surroundings onto one’s physical body is a direct manifestation of systemic racism on an individual, in this case resulting in PTB.

Racially/ethnically concentrated neighborhoods, unfairly disadvantaged through past and present policies, sustain health inequities of those who reside there at no fault of their own. Tackling this inequity means addressing racial/ethnic segregation. Disadvantaged neighborhoods experience inefficient public transportation; lower access to health care, affordable nutritious food, and safe places for physical activity; lower safety and higher air pollution; and lower-quality education and job opportunities. The findings support policies that prevent the gentrification-related displacement of POC in diverse neighborhoods. Confronting these systemic determinants of health is a pathway toward decreasing PTB and building long-term health equity.

## Data Availability

Data can be made available upon request.

## Code Availability

Available upon request.
